# Oncopacket: integration of cancer research data using GA4GH phenopackets

**DOI:** 10.1093/bioinformatics/btaf546

**Published:** 2025-09-29

**Authors:** Michael Sierk, Daniel Danis, Sujay Patil, Nobal Kishor, Rajdeep Mondal, Abhishek Jha, Qingrong Chen, Chunhua Yan, Monica Munoz-Torres, Daoud Meerzaman, Peter N Robinson, Justin T Reese

**Affiliations:** Center for Biomedical Informatics & Information Technology, National Cancer Institute (NCI) Bethesda, MD 20850, United States; Berlin Institute of Health at Charité Universitätsmedizin Berlin, Berlin 10117, Germany; The Jackson Laboratory for Genomic Medicine, Farmington, CT 06032, United States; Division of Environmental Genomics and Systems Biology, Lawrence Berkeley National Laboratory (LBNL), Berkeley, CA, 94720, United States; Elucidata, San Francisco, CA 94114, United States; Elucidata, San Francisco, CA 94114, United States; Elucidata, San Francisco, CA 94114, United States; Center for Biomedical Informatics & Information Technology, National Cancer Institute (NCI) Bethesda, MD 20850, United States; Center for Biomedical Informatics & Information Technology, National Cancer Institute (NCI) Bethesda, MD 20850, United States; Department of Biomedical Informatics, University of Colorado Anschutz Medical Campus, Aurora, CO 80045, United States; Center for Biomedical Informatics & Information Technology, National Cancer Institute (NCI) Bethesda, MD 20850, United States; Berlin Institute of Health at Charité Universitätsmedizin Berlin, Berlin 10117, Germany; The Jackson Laboratory for Genomic Medicine, Farmington, CT 06032, United States; Division of Environmental Genomics and Systems Biology, Lawrence Berkeley National Laboratory (LBNL), Berkeley, CA, 94720, United States

## Abstract

**Summary:**

Lack of data integration remains a significant impediment to cancer research, and many analyses still require customized software to transform and prepare cancer data. We describe a software package to harmonize genetic and clinical cancer data into the GA4GH Phenopacket schema, an ISO standard for representing clinical case data. We integrated demographic, mutation, morphology, diagnosis, intervention, and survival data using case data from the National Cancer Institute for 12 cancer types. The Phenopacket standard provides a foundation for downstream use, including sophisticated statistical and AI/ML analyses. We demonstrate fitness for purpose by using the integrated data to recapitulate a known association between mutations in the gene encoding isocitrate dehydrogenase 1 and survival time in brain cancer patients.

**Availability and implementation:**

Source code is freely available at: https://github.com/monarch-initiative/oncopacket (archived at 10.5281/zenodo.15353125).

## 1 Introduction

Cancer is a leading cause of mortality worldwide, with 24.5 million cases and at least 9.6 million deaths annually (Global Burden of Disease Cancer Collaboration *et al.* 2019). While abundant genomic, proteomic, demographic and clinical data is available for cancer research, integration of data remains a challenge; data is provided by several different consortia via many separate data commons and using different data models (Noor *et al.* 2015, Grossman *et al.* 2016, Learned *et al.* 2019).

Cancer data is currently represented using heterogeneous terminologies and data schemas, which makes it difficult to exchange data and often requires each individual dataset to be prepared for analysis using customized scripts or tools. The Global Alliance for Genomics and Health (GA4GH) Phenopacket schema can provide a Rosetta Stone to streamline data exchange and serve as a foundation for many types of statistical and machine learning analysis. The Phenopacket schema supports the exchange of computable longitudinal case-level phenotypic information for diagnosis of, and research on, all types of disease, including Mendelian and complex diseases, cancers, and infectious diseases (Jacobsen *et al.* 2022). Each phenopacket characterizes an individual person or biosample, linking that individual to detailed phenotypic descriptions, genetic information, diagnoses, and treatments (http://phenopackets.org).

Just as the adoption of the Variant Caller Format (VCF) for variant annotation enabled widespread sharing of genomic variant data and the development of software tools for analyzing such data, the GA4GH Phenopacket schema aspires to be similarly transformative for genome analysis using phenotype data. The multiple providers of phenotypic data include patients and clinicians and convey data via a variety of mechanisms, including clinical notes and electronic health records, interfaces such as FHIR (Ayaz *et al.* 2021), app-based entry, and mobile activity monitoring devices. The Phenopacket schema acts as a common model that can capture data from many sources with a unified software representation that can, in turn, be used by multiple receivers of the phenotypic information, including journals, databases, registries, and clinical laboratories. We anticipate that the Phenopacket schema will encourage the development of a collection of software for the analysis of genomic data in the context of clinical information that will accelerate innovation and discovery (Danis *et al.* 2023, GA4GH 2024a). Genomic data will become ever more important in translational research and clinical care in the coming years and decades. The Phenopacket schema represents a standard for capturing clinical data and integrating it with genomic data that will help to obtain the maximal utility from this data for understanding disease and developing precision medicine approaches to therapy. A detailed example of how to construct a phenopacket to represent the clinical course of a child with retinoblastoma is found in Ladewig *et al.* (2023).

A number of databases have adopted the Phenopacket standard to represent the clinical data of individuals in the context of rare-disease genomics (European Genome-phenome Archive), registries (European Joint Programme on Rare Diseases and Western Australian Register of Developmental Anomalies), biosamples (EMBL-EBI BioSamples database), and biobanks (the Japanese Agency for Medical Research and Development Tohoku Medical Megabank project and National Center Biobank Network). Here we illustrate the application of the Phenopacket standard to large-scale cancer data from the National Cancer Institute (NCI).

The Cancer Research Data Commons (CRDC) is a cloud-based infrastructure providing public and controlled-access to multiple large-scale cancer datasets that can be analyzed without download using the NCI Cloud Resources or as downloadable data for an individual researcher to explore using their own resources (Hinkson *et al.* 2017, Wang *et al.* 2024). It consists of six data commons: the Genomic Data Commons (GDC), the Proteomic Data Commons (PDC), the Imaging Data Commons (IDC), the Integrated Canine Data Commons (ICDC), the Cancer Data Service (CDS), and the Clinical and Translational Data Commons (CTDC), along with various services and tools. Each data commons uses specific formats, terminology, and data models that present barriers to cross-commons data harmonization and usage as well as integration with non-CRDC data. As described by Jill Barnholtz-Sloan (Barnholtz-Sloan 2022), these barriers need to be removed in order to maximize the impact of the vast amounts of data collected: “A national cancer data ecosystem, outlined as a priority for the original Cancer Moonshot … could one day unite these valuable, yet disparate, initiatives, as well as new initiatives being planned, under a single publicly available system.”

**Figure 1.**
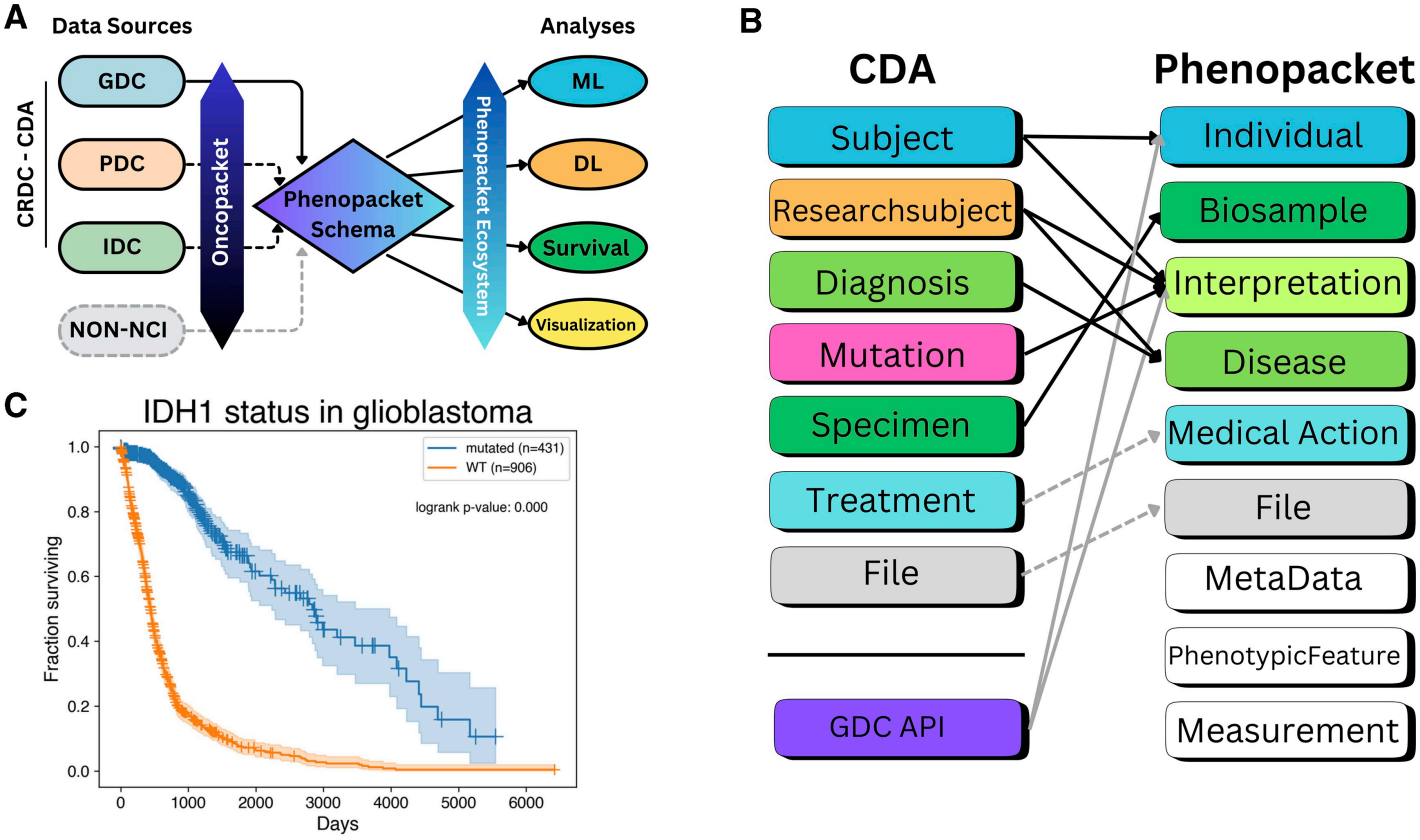
Transforming and analyzing cancer data using Oncopacket. (A) The role of Oncopacket data transformation. The package extracts data from the CRDC via the Cancer Data Aggregator interface. Dashed lines represent data sources not fully mapped yet. Further efforts will build on the existing code base to add mappers from other non-NCI data sources. (B) Illustration of the fields extracted from CDA and how they are mapped to the Phenopacket schema elements. (C) A Kaplan–Meier plot of a brain cancer cohort with and without IDH1 mutations generated from a cohort of Phenopackets produced by Oncopacket.

While the CRDC’s efforts to collect, harmonize, and provide accessible cancer data are essential, encoding CRDC data using the Phenopacket schema adds further value by facilitating integration with external data sources and tools (Fig. 1A). Converting CRDC data to the Phenopacket schema will benefit both sides: the phenopacket ecosystem will gain large amounts of data and new tools, and the CRDC ecosystem will gain a valuable integration pathway with other data sources. We describe here a pilot project demonstrating this conversion and the subsequent application to a simple clinical analysis. Future development will incorporate more data elements from CRDC as well as data from other external data sources.

## 2 Methods and results

We used the Cancer Data Aggregator Python library (Brady *et al.* 2024, *Cancer Data Aggregator 2024*) to extract cohorts from CRDC data and the Phenopackets Python library (GA4GH 2024b) to generate a phenopacket for each subject. In general, the fields mapped from CRDC are those that we judged to be the most important elements of the Phenopacket schema for likely downstream use cases (demographic data, diagnosis, mutation, phenotypic features, and vital status). For various reasons, some data elements, such as genome variant, variant consequence, cancer stage, and vital status, are retrieved directly via the Genomic Data Commons API (Grossman *et al.* 2016). (For example, cancer stage is recorded in four different fields in GDC but the diagnoses.tumor_stage field, which CDA uses, is not populated, so we used ajcc_pathologic_stage from GDC.) Oncopacket includes Python classes for individuals, diseases, and mutations that are combined to produce phenopackets. It also includes Python classes that extract data for biosamples, diseases, individuals, medical actions, and mutations from the CDA.

Oncopacket software accurately represents existing data from the upstream source, and does not impute missing records; we consider imputation of missing data an optional downstream task depending on use case. Oncopacket provides a suite of unit tests to ensure that data is faithfully transformed into phenopackets. In addition, Oncopacket provides Jupyter notebooks that allow users to characterize their cohorts and perform basic quality control, for example by plotting demographic data (sex, age, etc.), age of disease onset, and incidence of various diseases (Fig. 1, available as supplementary data at *Bioinformatics* online).

### 2.1 Mapping CRDC fields to the Phenopacket schema

Figure 1B depicts the mapping that Oncopacket performs between the CDA tables and the Phenopacket elements. Some components map directly, such as *Specimen* to *Biosample*, but many require combining information from multiple CDA tables. For example, constructing the Phenopacket *Disease* message data model requires fields from the *Diagnosis* and *Researchsubject* tables from CDA, joined on *researchsubject_id*. (In CDA a *researchsubject* can be either an individual patient or a sample from the patient.) We use the *primary_diagnosis* term from the *Diagnosis* table and the *primary_diagnosis_condition* and *primary_diagnosis_site* terms from the *Researchsubject* table to map to the corresponding NCI Thesaurus term. Terms were mapped once using Elucidata’s Polly, an LLM-based harmonization engine, to generate the disease ontology terms that are used during runtime. The mappings are available in the Github repo and will be updated periodically as needed. Polly uses a retrieval augmented generation (RAG) strategy to prevent hallucination. For each NCIt concept, we concatenate its preferred name, synonyms, and definition into a single string and generate one 768D embedding with a SAPBERT model fine-tuned for biomedical entity normalization. When a term (with site context) is queried, the seven closest candidates are retrieved by cosine similarity, and these candidates plus context are sent to GPT-4. Guided by Tree-of-Thought/ReAct prompting, the model reasons over the options and returns the single NCIt term that best fits the query. Accuracy was benchmarked against 850 gold standard terms independently annotated by four domain experts in biomedical ontology mapping. A prediction counts as correct only when it exactly matches the experts’ label. The engine achieved 98% exact-match (833/850) and 99.4% top-3 accuracy.

The modular code can be extended for use with other data sources, provided the appropriate code is written to convert the data into the Phenopacket format. Specifically, new Python modules can be added for new sources. Each module will contain source-specific logic for extracting, transforming, and loading data into the Oncopacket data model.

We have developed Python notebooks to demonstrate how to ingest phenopackets, perform simple analyses, and plot the results. Detailed documentation for Oncopacket is available at https://monarch-initiative.github.io/oncopacket/.

### 2.2 Generation and analysis of cohorts

We used Oncopacket to generate several cohorts for 12 different tissues, available as a Zenodo dataset (https://doi.org/10.5281/zenodo.14610228). The scripts to generate these cohorts are provided in the scripts directory and can be modified to produce other cohorts of interest. Table 1, available as supplementary data at *Bioinformatics* online shows the number of phenopackets generated and the time to generate them for each cohort, ranging from 3 min, 4 s to generate 324 phenopackets in the bone cohort to 47 min, 18 s to generate 5449 phenopackets in the lung cohort. The scripts do not require >1 GB of memory to run. Most of the time is used to complete the API calls to the GDC.

We created Python notebooks illustrating how to (i) extract data from a cohort of Oncopacket-generated phenopackets, (ii) generate summary plots of the cohort, and (iii) perform survival analysis using the lifelines package (Davidson-Pilon 2019).

To illustrate the utility of Oncopacket for downstream analyses, we generated a brain cancer cohort and produced Kaplan–Meier curves for individuals with and without an IDH1 mutation, recapitulating the well-known increased survival time of patients with IDH1 mutations (Nobusawa *et al.* 2009) (Fig. 1C). We also provide summary bar charts of a lung cancer cohort in Fig. 1, available as supplementary data at *Bioinformatics* online.

## 3 Discussion

Despite significant progress over the past decade, the integration of cancer data remains a major obstacle for cancer researchers. While several existing initiatives, such as ICGC ARGO (ICGC ARGO—Home 2025), NCDB (National Cancer Database 2025), TCGA [The Cancer Genome Atlas Program (TCGA) – NCI 2025], VICC (Standardizing Cancer Variant Knowledge to Enable Precision Oncology 2025), and the NCI Data Commons (Hinkson *et al.* 2017) have made strides in standardizing cancer data, directly using data from these sources for research is still challenging. To address this, Oncopacket builds upon these advances by aligning data to the interoperable GA4GH Phenopacket schema. This alignment provides a crucial pathway around the integration impediment, enabling direct compatibility with a growing ecosystem of downstream tools.

Currently, Oncopacket aligns with Phenopacket schema version 2, an ISO standard anticipated to offer stable, long-term support. Should future versions of the Phenopacket schema be released, Oncopacket updates will be guided by the adoption patterns and compatibility needs of key downstream tools within the phenopacket ecosystem.

This growing software ecosystem aligned with phenopackets includes tools for tasks like prioritizing genome variants for human disease (Smedley *et al.* 2015), comparing individuals based on phenotypic features (Leist *et al.* 2024), performing sophisticated statistical testing of genotype-phenotype correlations (GPSEA, manuscript in preparation), and collecting deeply phenotyped case data from scientific literature (Danis *et al.* 2025). While further work is needed to more comprehensively map CRDC and other data sources to the Phenopacket schema, Oncopacket provides the foundation to facilitate similar downstream integration and analysis of cancer data from NCI and other sources.

## Supplementary Material

btaf546_Supplementary_Data

## Data Availability

Phenopackets for 23650 individuals from 12 cancer types, 7816 of which have mutational data (average 80 variants affecting 62 unique genes per patient), are available as a Zenodo dataset: https://doi.org/10.5281/zenodo.14610228. An example of plots summarizing a cohort of phenopackets is available in the online supplement.
