# Supplementary material for: Oncopacket: integration of cancer research data using GA4GH phenopackets
Source: Bioinformatics. 2025 Sep 29;41(10):btaf546. doi: 10.1093/bioinformatics/btaf546 (PMC12516310; doi:10.1093/bioinformatics/btaf546)
Supplement: btaf546_Supplementary_Data [file btaf546_supplementary_data.docx]

**Supplementary Data**

**
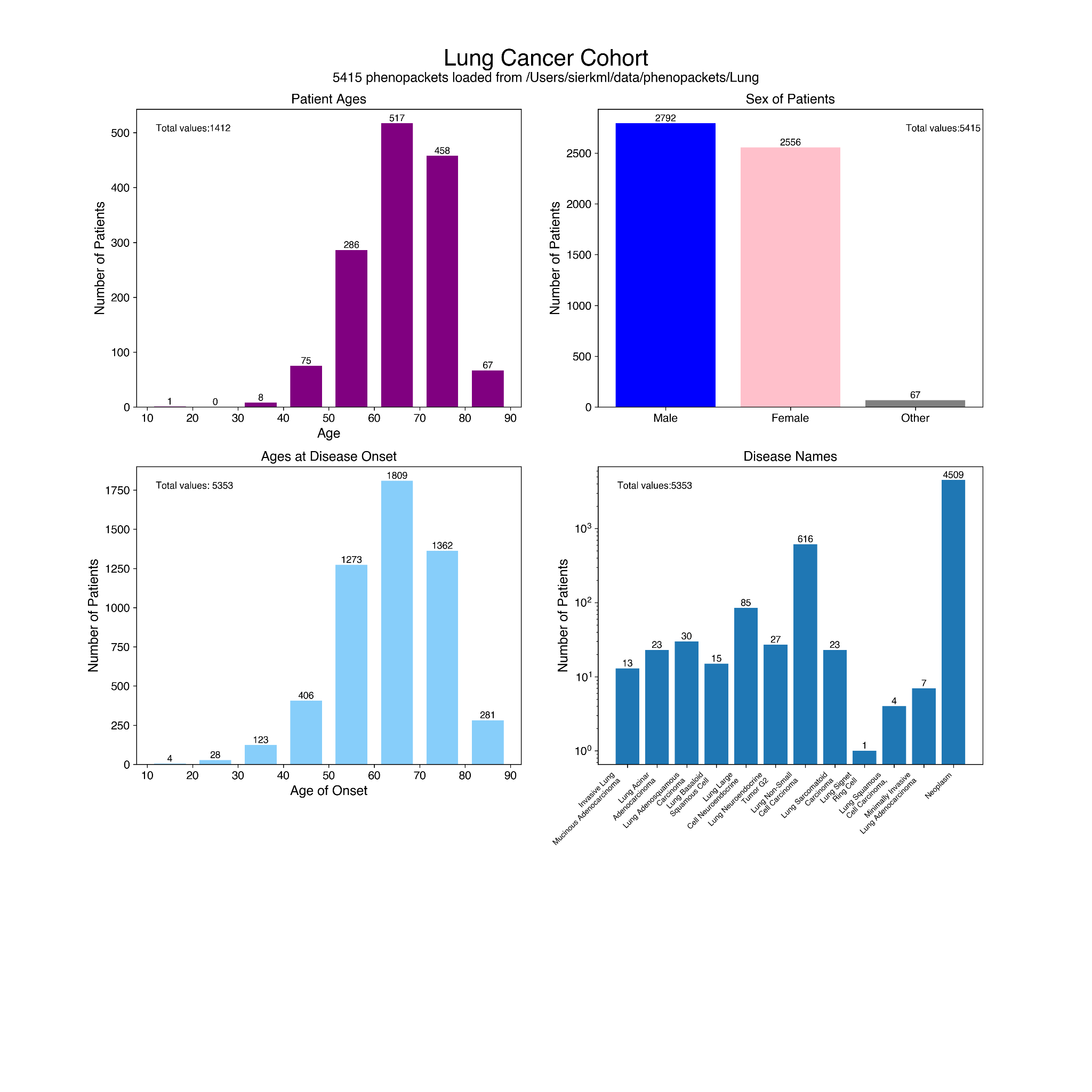
**

**Supplementary Figure 1.** Summary bar charts of a lung cancer cohort produced by Oncopacket.

| **Tissue** | **Number of Phenopackets** | **Time (min:sec)** |
| --- | --- | --- |
| Bone | 324 | 3:04 |
| Brain | 1423 | 14:36 |
| Breast | 4120 | 28:01 |
| Cervix | 1982 | 15:57 |
| Colon | 3013 | 20:16 |
| Kidney | 2427 | 16:27 |
| Liver | 858 | 8:08 |
| Lung | 5449 | 47:18 |
| Pancreas | 1215 | 8:54 |
| Skin | 842 | 10:50 |
| Stomach | 890 | 7:53 |
| Thyroid | 1125 | 8:10 |

**Supplementary Table 1.** Number of and time to create the twelve example tissue-based phenopacket cohorts on an Apple MacBook M2 with 16 GB of memory.

**Supplementary Methods:**

**Polly: LLM-based harmonization tool**

We employed Polly, an LLM-based harmonization tool for performing mappings to ontology terms. Mappings were performed once, and were used during runtime. A more detailed description of Polly follows below.

For any given mention, our system first employs a deterministic algorithm (SapBERT + TF-IDF) to retrieve the top 10 candidate IDs from a static, version-controlled ontology. The LLM is then constrained to only rank or reject this pre-selected list. This architecture ensures that an LLM upgrade can, at most, re-order the candidates; it is incapable of introducing novel or out-of-scope identifiers.

No new LLM version is adopted without passing a stringent quality benchmark. The candidate model must match or exceed the current top-1 accuracy baseline on our 850-mention, expert-curated test set for every distinct entity. Failure to meet this standard for even a single entity type results in the immediate rejection of the upgrade.

We enforce a systematic monitoring protocol after deployment. Every production run logs a complete set of immutable identifiers (model hash, code commit, ontology checksum). A consolidated performance report is then automatically generated on a fixed 30-day schedule for review by our human curation team, ensuring both automated traceability and expert vigilance against any potential performance drift.

Repeatability of LLM responses

We guarantee a deterministic starting point for every analysis. Our ontology files are content-addressed using SHA-256, ensuring data integrity, and the candidate retrieval process operates with a fixed random seed for algorithmic consistency. As a result, the same input mention will invariably produce the identical candidate list across every run.

To eliminate any randomness in the reasoning stage, we set the sampling parameters to temperature=0 and top_p=1, we compel the model to always select the single most probable output, guaranteeing that a given input and prompt will produce an identical result on every execution.

In a controlled reproducibility experiment, we processed the same 850-mention golden dataset 10 times under identical conditions and achieved 100% output consistency, every mention mapped to the same ontology ID across all runs.

Preventing hallucination

We implement a Retrieval-Augmented Generation (RAG) pattern to prevent hallucination. For each candidate, the LLM is provided the definition and synonyms retrieved from the ontology. The model’s output is therefore strictly constrained. It can only select an ID from the pre-vetted candidate list or return the literal string “None.” This prevents the invention of fabricated or irrelevant identifiers.

Our prompt architecture enforces a multi-step reasoning framework to make every decision transparent and logically sound, drawing from established research.

The model must first generate and evaluate multiple possible interpretations for each term, a method of structured exploration inspired by "Tree of Thoughts ([[2305.10601] Tree of Thoughts: Deliberate Problem Solving with Large Language Models](https://arxiv.org/abs/2305.10601))" that improves complex problem-solving.

For its final selection, the model must generate a step-by-step "Chain of Thought ([[2201.11903] Chain-of-Thought Prompting Elicits Reasoning in Large Language Models](https://arxiv.org/abs/2201.11903))," an approach that elicits more robust reasoning and makes the entire decision pathway auditable.

This process concludes with the model outputting numerical confidence and context_influence scores for every candidate it considered, providing a clear audit trail of its evaluation before selecting the final answer.
